# Ru complexes of Hoveyda–Grubbs type immobilized on lamellar zeolites: activity in olefin metathesis reactions

**DOI:** 10.3762/bjoc.11.225

**Published:** 2015-11-04

**Authors:** Hynek Balcar, Naděžda Žilková, Martin Kubů, Michal Mazur, Zdeněk Bastl, Jiří Čejka

**Affiliations:** 1 J. Heyrovský Institute of Physical Chemistry, Academy of Sciences of the Czech Republic, v.v. i. Dolejškova 2155/3, 182 23 Prague 8, Czech Republic

**Keywords:** Hoveyda–Grubbs type catalyst, hybrid catalysts, lamellar zeolites, non-covalent immobilization, olefin metathesis

## Abstract

Hoveyda–Grubbs type catalysts with cationic tags on NHC ligands were linker-free immobilized on the surface of lamellar zeolitic supports (MCM-22, MCM-56, MCM-36) and on mesoporous molecular sieves SBA-15. The activity of prepared hybrid catalysts was tested in olefin metathesis reactions: the activity in ring-closing metathesis of citronellene and *N*,*N*-diallyltrifluoroacetamide decreased in the order of support MCM-22 ≈ MCM-56 > SBA-15 > MCM-36; the hybrid catalyst based on SBA-15 was found the most active in self-metathesis of methyl oleate. All catalysts were reusable and exhibited low Ru leaching (<1% of Ru content). XPS analysis revealed that during immobilization ion exchange between Hoveyda–Grubbs type catalyst and zeolitic support occurred in the case of Cl^−^ counter anion; in contrast, PF_6_^−^ counter anion underwent partial decomposition.

## Introduction

Immobilization of Ru alkylidene complexes (Grubbs and Hoveyda–Grubbs type catalysts) on siliceous supports represents a successful way to highly active, selective, and reusable metathesis catalysts [1–4]. Mesoporous molecular sieves (MCM-41, MCM-48, SBA-15), with large BET areas and pore volumes, proved to be very suitable supports, due to easy attachment of bulky organometallic complexes onto silica surface ensuring rapid diffusion of reactants to the active catalytic sites [5–12]. Several strategies of immobilization have been developed [1,5,13]; most of them are based on surface modification by specially designed linkers providing covalent bond linkage between the support and Ru complex. Hoveyda–Grubbs type catalysts are also capable of direct (linker-free) immobilization by means of non-covalent interactions [8,14–20]. Although the character of this interaction is not completely clear, they are firm enough to ensure low Ru leaching and catalyst reusability.

Recently, we reported Hoveyda–Grubbs type catalysts bearing quaternary ammonium tag on NHC ligand (HGIIN^+^X, where X = Cl^−^, I^−^, PF_6_^−^, or BF_4_^−^) and their immobilization on silica, and mesoporous molecular sieves MCM-41 and SBA-15 [21]. XPS analysis revealed that complexes were attached to the surface by non-covalent interactions and both cationic and anionic parts were present on the surface. The hybrid catalysts prepared were active in RCM of 1,7-octadiene and (−)-β-citronellene; HGIIN^+^Cl^−^ on SBA-15 (HGIIN^+^Cl^−^/SBA-15) was the most active (TON up to 16000 in RCM of citronellene). HGIIN^+^Cl^−^/SBA-15 proved its versatility in RCM, enyne metathesis, metathesis of methyl oleate, and cross-metathesis of electron deficient methyl acrylate with various co-substrates. The catalyst was reusable and Ru leaching was very low, not only in toluene (Ru content in product <10 ppm in most cases) but also in polar solvents (ethyl acetate, dichloromethane, leaching about 1% of Ru content in catalyst). A similar ammonium-tagged Hoveyda–Grubbs type catalyst with sterically enlarged NHC ligand supported on SBA-15 exhibited high stability and was effective in flow reactions [22].

**Figure 1:**
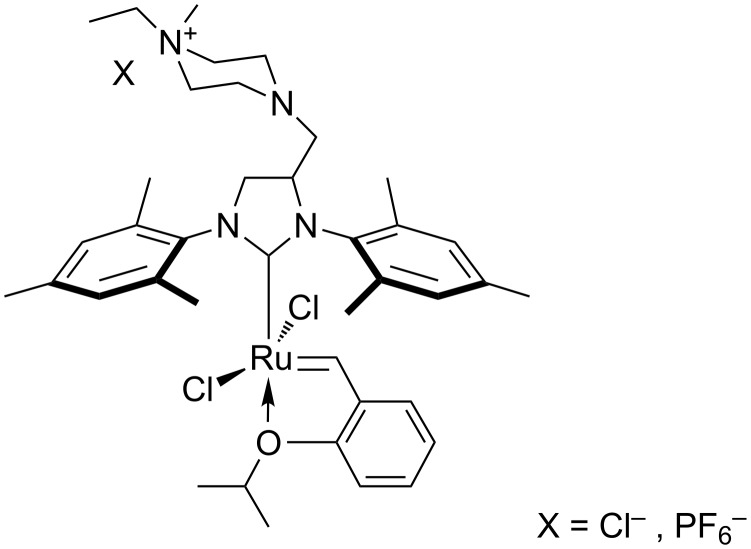
Hoveyda–Grubbs type catalysts used for immobilization.

According to our knowledge, zeolites have not been considered as perspective supports for the immobilization of Ru metathesis catalysts due to small diameters of their pores (<1 nm) not allowing to anchor appropriate alkylidene complexes in the channel system and to ensure accessibility of catalytic centers by reactants. However, new methods for the preparation of lamellar (also called two-dimensional) zeolite with high surface area and layered structure have been developed [23] and such zeolites offer the possibility of their modifications with organometallic moieties in a similar way as mesoporous molecular sieves. Limbach et al. [20] used MWW material as a support for Ru heterogeneous catalyst for cyclooctene oligomerization, however, its activity was rather low. In this article we discuss the immobilization of HGIIN^+^Cl^−^ and HGIIN^+^PF_6_^−^ (Figure 1) on zeolitic supports having MWW structure: MCM-22 (three-dimensional), MCM-56 (unilamellar), and MCM-36 (pillared) and the activity of corresponding catalysts (i) in RCM of (−)-β-citronellene and *N*,*N*-diallyl-2,2,2-trifluoroacetamide (DAF), and (ii) in self-metathesis and cross-metathesis of methyl oleate.

Lamellar (two dimensional) zeolites represent a subgroup of zeolitic materials, in which one of the dimension of the crystals is usually limited to 2–3 nm and is around one unit cell [24–25]. Depending on the structure of the prepared zeolite, the individual zeolitic layers exhibit or do not exhibit micropore character. Two dimensional zeolites are usually prepared by a bottom-up hydrothermal synthesis [26]; recently also a top-down approach from germanosilicate zeolite UTL was reported [27]. The latter approach utilizes chemically selective hydrolysis of Ge–O bonds to form layers from three-dimensional zeolites [28]. Generally, two-dimensional zeolites possess BET areas above 500–600 m^2^/g, which is comparable with mesoporous molecular sieves. The surface of two-dimensional zeolites can be modified with various organic ligands to induce adsorption or catalytic functionalities [29–30]. The detailed structures of zeolites MCM-22, MCM-36 and MCM-56 used as supports in this work are depicted in [31–32].

## Results and Discussion

### Hybrid catalyst preparation and characterization

Immobilization of HGIIN^+^X complexes proceeded smoothly by mixing their solutions with dry supports at room temperature. In the case of HGIIN^+^Cl^−^ and MCM-22, MCM-56, and SBA-15, the immobilization was nearly quantitative (97–99% of Ru was attached to the support, see Experimental). However, in other cases (HGIIN^+^Cl^−^ + MCM-36 and HGIIN^+^PF_6_^−^ + MCM-22) only part of Ru submitted for immobilization was captured on the support under condition applied. In this way, hybrid catalysts HGIIN^+^Cl^−^/MCM-22 (1.1 wt % Ru), HGIIN^+^Cl^−^/MCM-56 (1.1 wt % Ru), HGIIN^+^Cl^−^/MCM-36 (0.7 wt % Ru), HGIIN^+^PF_6_^−^/MCM-22 (0.9 wt % Ru), and HGIIN^+^Cl^−^/SBA-15 (1.2 wt % Ru) were prepared.

Table 1 shows textural parameters of zeolitic supports and corresponding hybrid catalysts. The attachment of Ru complex brought about a significant decrease in *S*_BET_ and pore volume. Especially, the micropore volume strongly decreased. Due to the molecular size of Hoveyda–Grubbs 2^nd^ generation catalyst (1.76 × 1.35 × 1.05 nm [15]) the molecules of HGIIN^+^Cl^−^ cannot penetrate into micropores of MCM-22 or MCM-56 zeolites. The decrease in the micropore volume may suggest that molecules of catalyst are located in the mouths of pores and block the access to the micropore system. X-ray diffraction patterns showed (Supporting Information File 1, Figures S1, S2, and S3) that original structure of the parent supports was preserved. As concerns HGIIN^+^Cl^−^/SBA-15, it was shown earlier [21] that the SBA-15 architecture was preserved; both *S*_BET_ and *V* values were reduced in comparison with the parent SBA-15 (from 739 m^2^/g and 1.15 cm^3^/g to 492 m^2^/g and 0.92 cm^3^/g, respectively) but the change in pore diameter was negligible (from 6.7 to 6.6 nm).

**Table 1:** Textural parameters of MCM-22, MCM-56, MCM-36, and corresponding hybrid catalysts.

Sample	*S*_BET_ (m^2^/g)	*S*_ext_^a^ (m^2^/g)	*V*_mic_^b^ (cm^3^/g)	*V*_total_^c^ (cm^3^/g)

MCM-22	504	121	0.174	0.429
HGIIN^+^Cl^−^/MCM-22	379	121	0.117	0.355
MCM-56	446	171	0.124	0.555
HGIIN^+^Cl^−^/MCM-56	157	119	0.015	0.324
MCM-36	658	564	0.041	0.364
HGIIN^+^Cl^−^/MCM-36	488	426	0.027	0.268

^a^External surface area, ^b^micropore volume (*t-*plot method), ^c^total pore volume at *p*/*p*_0_ = 0.95, for evaluation of *S*_BET_, the interval of *p*/*p*_0_ = 0.05–0.20 was used.

The stoichiometry of the studied catalyst samples resulting from the XPS analysis is summarized in Table 2. A good agreement between the chemical composition of the neat compounds calculated from the integrated intensities of photoelectron spectra and their nominal stoichiometry was observed. For HGIIN^+^Cl^−^/SBA-15 the atomic ratio Cl/Ru = 3 indicates both cationic and anionic parts of the parent complex were present in the hybrid catalysts, as shown earlier [21]. In contrast to that, the atomic ratio Cl/Ru = 1.9 for HGIIN^+^Cl^−^/MCM-22 may indicate that Cl^−^ remained in a liquid phase as NaCl. For HGIIN^+^PF_6_^−^/MCM-22 catalyst, the results suggest that reduction of the PF_6_ anion to the PF_3_ species took place in immobilized compound. In addition to it, the decrease in Cl/Ru atomic ratio to 1.3 (1.4) may indicate change in the number of Cl ligands in the coordination sphere of Ru (at least a part of catalyst molecules was affected). The low concentration of the Ru complex in HGIIN^+^PF_6_^−^/MCM-22 did not allow obtaining any detailed information.

**Table 2:** Atomic concentration ratios of N, F, Cl, and P to Ru determined from XP spectra for neat HGIIN^+^X (X = Cl^−^, PF_6_^−^) and hybrid catalysts HGIIN^+^Cl^−^/MCM-22, HGIIN^+^Cl^−^/SBA-15, and HGIIN^+^PF_6_^−^/MCM-22. (For HGIIN^+^PF_6_^−^/MCM-22 catalyst the results obtained on two independent sample preparations are displayed demonstrating the reproducibility.)

Sample	N	Cl	F	P

HGIIN^+^Cl^−^	4.2	3.0	0	0
HGIIN^+^Cl^−^/MCM-22	4.1	1.9	0	0
HGIIN^+^Cl^−^/SBA-15	4.0	3.0	0	0
HGIIN^+^PF_6_^−^	3.8	1.8	6.2	1.0
HGIIN^+^PF_6_^−^/MCM-22	4.2 4.0	1.3 1.4	2.9 3.2	1.2 0.85

### Catalyst activity in ring-closing metathesis

**Scheme 1:**
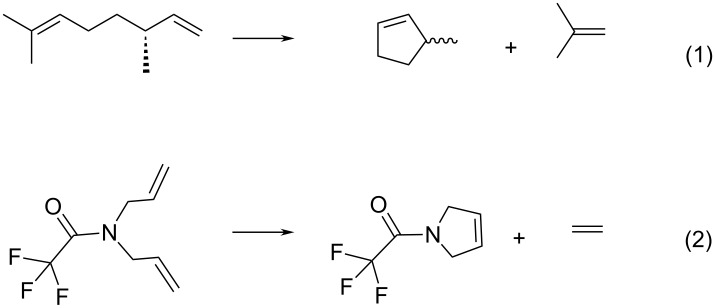
RCM of (−)-β-citronellene (1) and *N*,*N*-diallyl-2,2,2-trifluoroacetamide (2).

**Figure 2:**
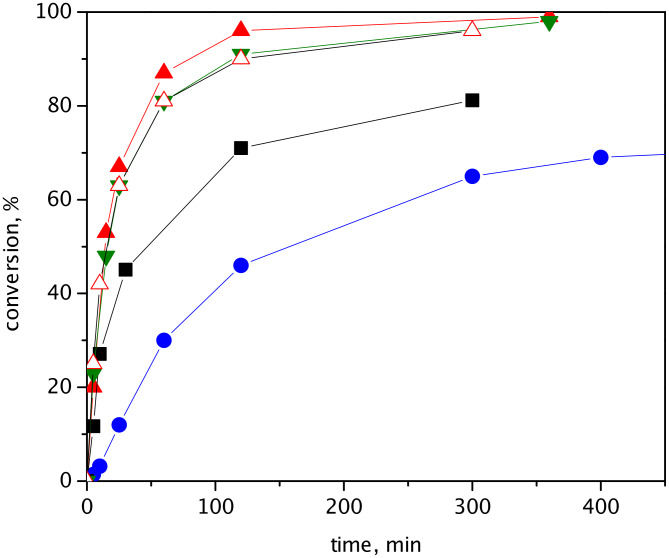
Conversion vs time dependence for RCM of (−)-β-citronellene over HGIIN^+^Cl^−^/MCM-36 (●), HGIIN^+^Cl^−^/SBA-15 (■), HGIIN^+^Cl^−^/MCM-22 (▲), HGIIN^+^PF_6_^−^/MCM-22 (∆), and HGIIN^+^Cl^−^/MCM-56 (▼). Toluene, 60 °C, molar ratio (−)-β-citronellene/Ru = 2000, *c*_citr_ = 0.15 mol/L.

Hybrid catalysts were tested in ring-closing metathesis (RCM) of (−)-β-citronellene and *N*,*N*-diallyl-2,2,2-trifluoroacetamide (DAF) (Scheme 1). Figure 2 shows conversion curves of RCM of (−)-β-citronellene over HGIIN^+^Cl^−^/MCM-22, HGIIN^+^Cl^−^/MCM-56, HGIIN^+^Cl^−^/MCM-36, HGIIN^+^PF_6_^−^/MCM-22, and HGIIN^+^Cl^−^/SBA-15 for comparison (data taken from ref [21] for the last catalyst). It is seen that the activities of HGIIN^+^Cl^−^/MCM-22, HGIIN^+^Cl^−^/MCM-56, and HGIIN^+^PF_6_^−^/MCM-22 were rather similar but significantly higher than that of HGIIN^+^Cl^−^/SBA-15. The initial TOFs (calculated from conversion at 5 min) were 4800 h^−1^, 5500 h^−1^, and 2800 h^−1^ for HGIIN^+^Cl^−^/MCM-22, HGIIN^+^Cl^−^/MCM-56, and HGIIN^+^Cl^−^/SBA-15, respectively, and also the conversion after 300 min was higher for HGIIN^+^Cl^−^/MCM-22 and HGIIN^+^Cl^−^/MCM-56 (98% and 97%, respectively) than for HGIIN^+^Cl^−^/SBA-15 (81%). It demonstrates the superiority of both HGIIN^+^Cl^−^/MCM-22 and HGIIN^+^Cl^−^/MCM-56 catalysts in this reaction, originating probably from a better accessibility of catalytic centers. The conversion curve for HGIIN^+^PF_6_^−^/MCM-22 was close to that for HGIIN^+^Cl^−^/MCM-22, in spite of changes of HGIIN^+^PF_6_^−^ structure in the course of immobilization as indicated by XPS. Surprisingly, conversions achieved with HGIIN^+^Cl^−^/MCM-36 were lower than those achieved with HGIIN^+^Cl^−^/MCM-22 and even with HGIIN^+^Cl^−^/SBA-15, despite the pillared character of the MCM-36 support. Selectivity was 100% in all cases: only methylcyclopentene and isobutene were found as reaction products by GC–MS. Enantioselectivity was not established.

**Figure 3:**
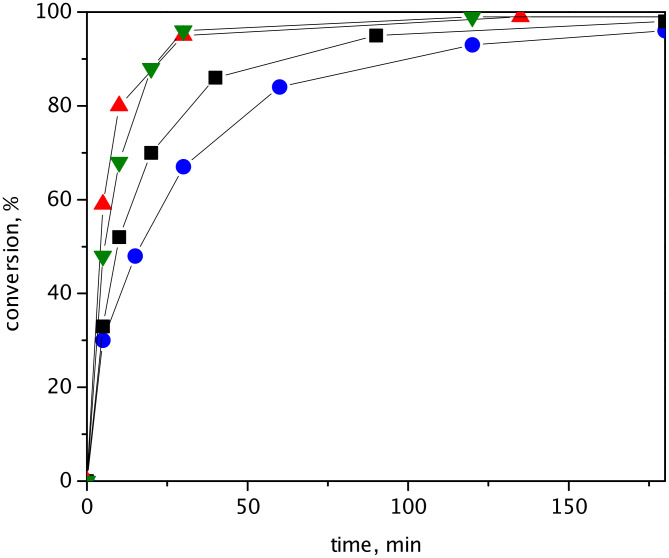
Conversion vs. time dependences for RCM of DAF over catalysts HGIIN^+^Cl^−^/MCM-22 (▲), HGIIN^+^Cl^−^/MCM-56 (▼), HGIIN^+^Cl^−^/SBA-15 (■), and HGIIN^+^Cl^−^/MCM-36 (●). Toluene, 30 °C, molar ratio DAF/Ru = 250, *c*_DAF_ = 0.15 mol/L.

Similar dependence of catalytic activity on the type of support was found for RCM of DAF (Figure 3). The initial TOFs (calculated from conversion at 5 min) decreased in the order: HGIIN^+^Cl^−^/MCM-22 (1770 h^−1^) > HGIIN^+^Cl^−^/MCM-56 (1440 h^−1^) > HGIIN^+^Cl^−^/SBA-15 (990 h^−1^) ≥ HGIIN^+^Cl^−^/MCM-36 (900 h^−1^). Final conversions (at 180 min) were in the interval from 96% to 99%. Similarly as for RCM of (−)-β-citronellene, hybrid catalysts HGIIN^+^Cl^−^/MCM-22 and HGIIN^+^Cl^−^/MCM-56 exhibited a higher activity than HGIIN^+^Cl^−^/SBA-15. Although initial TOF for HGIIN^+^Cl^−^/SBA-15 and for HGIIN^+^Cl^−^/MCM-36 were close to each other; further progress of conversion curves indicated lower activity of HGIIN^+^Cl^−^/MCM-36 in comparison with HGIIN^+^Cl^−^/SBA-15. The selectivity to *N*-(2-trifluoroacetyl)-2,5-dihydropyrrole was 100%.

**Figure 4:**
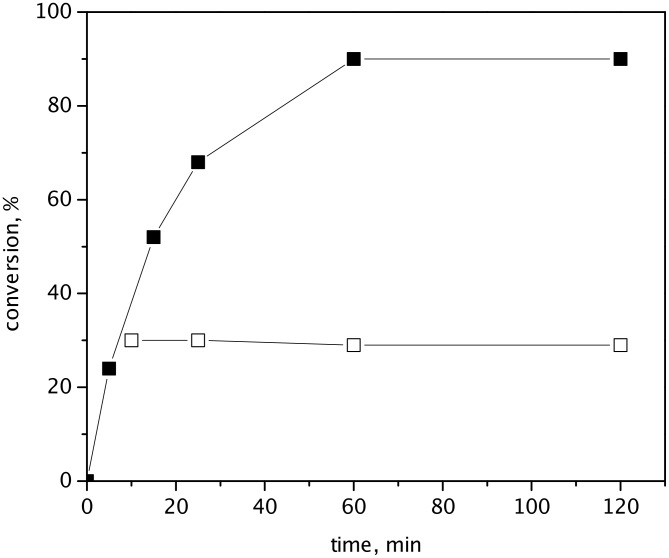
Splitting test for HGIIN^+^Cl^−^/MCM-56 in RCM of (−)-β-citronellene. Toluene, 60 °C, molar ratio (−)-β-citronellene/Ru = 2000, *c*_citr_ = 0.15 mol/L. Heterogeneous system (■), filtrate (□).

Catalyst leaching and reusing were studied in RCM of (−)-β-citronellene. Figure 4 shows a splitting test [33] for HGIIN^+^Cl^−^/MCM-56. 10 min after the beginning of the reaction, a half of the liquid phase was filtered off into a parallel reactor further kept under the same reaction temperature. Metathesis reaction continued in the heterogeneous system only, which evidences no leaching of catalytically active species into the liquid phase. Ru leaching determined by elemental analysis in the reaction mixture after finishing the reaction was 0.3%, 0.1%, and 0.6% of starting amount of Ru in catalyst for HGIIN^+^Cl^−^/MCM-22, HGIIN^+^Cl^−^/MCM-56, and HGIIN^+^Cl^−^/MCM-36, respectively. These values correspond to 1.2, 0.4, and 2.2 ppm of Ru in the products, which is considerably lower than the Ru content in drugs recommended by the European Medicines Agency in 2007 (10 ppm for oral exposure) [34].

Results of HGIIN^+^Cl^−^/MCM-22 reusing are displayed in Table 3. The catalyst was used 5 times without any decrease in the conversion. Due to the very low Ru leaching level (only 0.3% of the original amount of Ru was found in the combined samples from runs 1 to 5), the conversion drop after the fifth run must be ascribed to the catalyst deactivation. The cumulative TON achieved in 7 runs was 1491. The results evidence very firm attachment of catalytically active species to the surface of zeolites and their good stability.

**Table 3:** HGIIN^+^Cl^−^/MCM-22 reusing in RCM of (−)-β-citronellene. Toluene, 60 °C, molar ratio (−)-β-citronellene/Ru = 1:250, *c*_citr_ = 0.15 mol/L, reaction time 2.5 h.

Run	1	2	3	4	5	6	7

Conversion	99.5%	99.5%	99.5%	99.5%	99.5%	72.5%	26.5%
Cumulative TON	249	498	746	995	1244	1425	1491

### Catalyst activity in self-metathesis and cross-metathesis of methyl oleate

**Figure 5:**
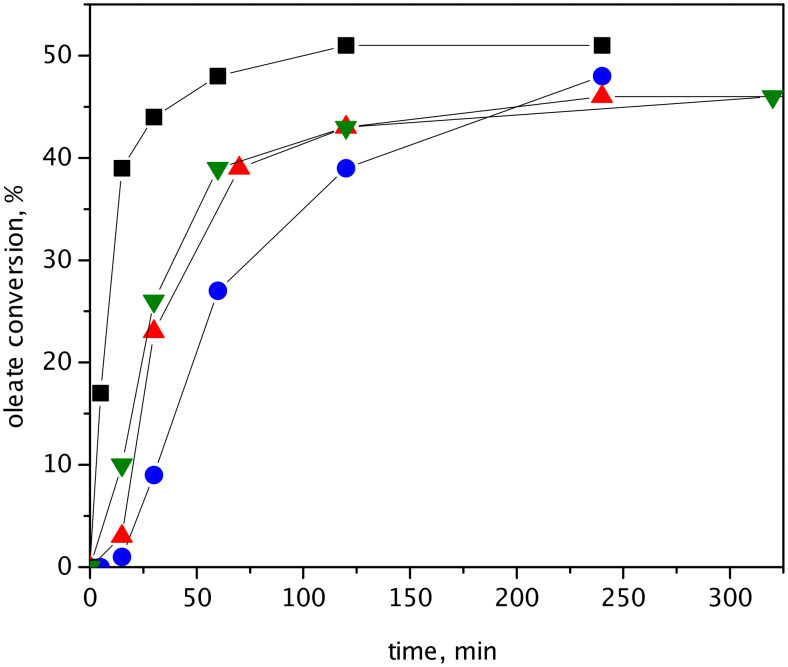
Self-metathesis of methyl oleate over HGIIN^+^Cl^−^/SBA-15 (■), HGIIN^+^Cl^−^/MCM-22 (▲), HGIIN^+^Cl^−^/MCM-56 (▼), and HGIIN^+^Cl^−^/MCM-36 (●). Toluene, 60 °C, molar ratio oleate/Ru = 250, *c*_ol_ = 0.15 mol/L.

Conversion curves for self-metathesis of methyl oleate over hybrid catalysts HGIIN^+^Cl^−^/MCM-22, HGIIN^+^Cl^−^/MCM-56, HGIIN^+^Cl^−^/MCM-36, and HGIIN^+^Cl^−^/SBA-15 in toluene at 60 °C are depicted in Figure 5. In contrast to RCM of (−)-β-citronellene and RCM of DAF, HGIIN^+^Cl^−^/SBA-15 turned out to be the most active catalyst. The catalytic activity decreased in the order HGIIN^+^Cl^−^/SBA-15 > HGIIN^+^Cl^−^/MCM-56 ≈ HGIIN^+^Cl^−^/MCM-22 > HGIIN^+^Cl^−^/MCM-36. Moreover, conversion curves for catalysts supported on zeolites exhibited an induction period repeatedly (very distinct for HGIIN^+^Cl^−^/MCM-22 and HGIIN^+^Cl^−^/MCM-36). This induction period became even more pronounced when the reaction temperature decreased to 30 °C and the activity gap between HGIIN^+^Cl^−^/SBA-15 on one side and HGIIN^+^Cl^−^/MCM-22 and HGIIN^+^Cl^−^/MCM-56 on the other side strongly increased (Supporting Information File 1, Figure S4). With HGIIN^+^Cl^−^ as a homogeneous catalyst no induction period was discernable at 60 °C (see [21]), however, the conversion curve at 30 °C (Supporting Information File 1, Figure S4) suggests a short induction period similar to the reaction with HGIIN^+^Cl^−^/SBA-15. In all cases, octadecene and dimethyl octadecendioate were the only reaction products.

**Scheme 2:**
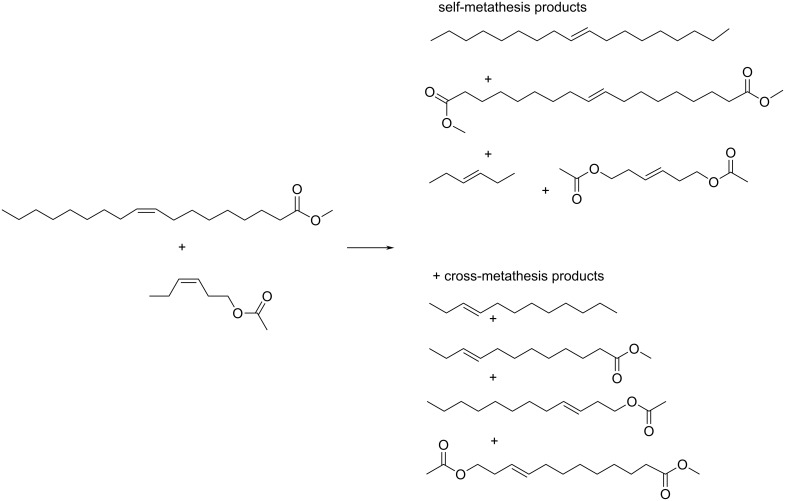
Cross-metathesis of methyl oleate with *cis*-3-hexenyl acetate.

**Figure 6:**
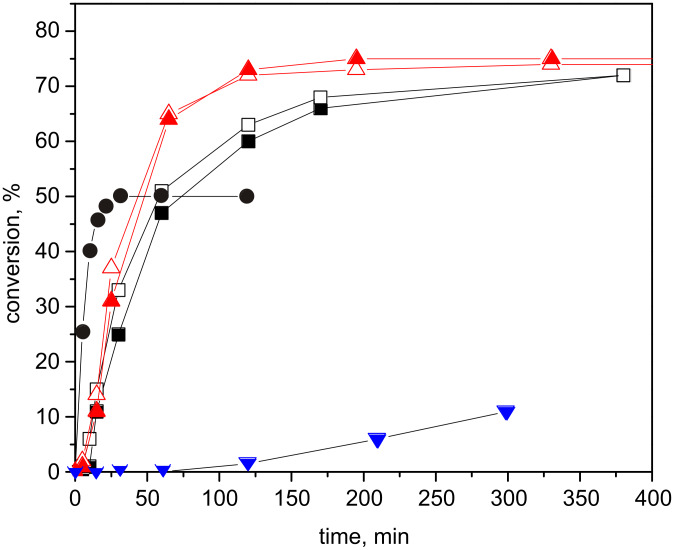
Conversion curves for CM of methyl oleate (full symbols) with *cis*-3-hexenyl acetate (open symbols) over HGIIN^+^Cl^−^/MCM-22 (▲,∆), and HGIIN^+^Cl^−^/SBA-15 (■,□) and for self-metathesis of oleate (▼) and self-metathesis of cis-3-hexenyl acetate (●) both with HGIIN^+^Cl^−^/MCM-22. Toluene, 30 °C, molar ratio methyl oleate/*cis*-3-hexenyl acetate/Ru = 250/250/1, *c*_ol_ = *c*_ac_ = 0.15 mol/L.

In order to elucidate the origin of the above mentioned difference in activity of HGIIN^+^Cl^−^/SBA-15 and HGIIN^+^Cl^−^/MCM-22, we performed a study of cross-metathesis (CM) of methyl oleate and *cis*-3-hexenyl acetate (Scheme 2) over these two catalysts. *cis*-3-Hexenyl acetate can be considered as a short-chain analogue of methyl oleate. Over both HGIIN^+^Cl^−^/SBA-15 and HGIIN^+^Cl^−^/MCM-22 in toluene at 30 °C, *cis*-3-hexenyl acetate reacted quickly, without any induction period, and with 100% selectivity to 3-hexene and 1,6-diacetoxy-3-hexene. The differences in the reaction rates for both catalysts were marginal (Figure S5, Supporting Information File 1). Splitting test for self-metathesis of *cis*-3-hexenyl acetate over HGIIN^+^Cl^−^/MCM-22 (Figure S6, Supporting Information File 1) evidenced no leaching of catalytically active species into the liquid phase, similarly to RCM of (−)-β-citronellene over HGIIN^+^Cl^−^/MCM-56. Figure 6 shows conversion curves for CM of methyl oleate with cis-3-hexenyl acetate (molar ratio 1:1) over both HGIIN^+^Cl^−^/MCM-22 and HGIIN^+^Cl^−^/SBA-15 together with conversion curves for self-metatheses of methyl oleate and *cis*-3-hexenyl acetate over HGIIN^+^Cl^−^/MCM-22. The induction period characteristic for self-metathesis of methyl oleate over HGIIN^+^Cl^−^/MCM-22 was minimized in CM to about 5 min for both catalysts. The reaction proceeded more quickly over HGIIN^+^Cl^−^/MCM-22 than over HGIIN^+^Cl^−^/SBA-15 (cf. 31% conversion of methyl oleate at 25 min over HGIIN^+^Cl^−^/MCM-22 vs 25% methyl oleate conversion at 30 min over HGIIN^+^Cl^−^/SBA-15). At the beginning of the reaction, the consumption of *cis*-3-hexenyl acetate prevailed over that of methyl oleate, however, approaching the equilibrium, the consumptions of both reactants were practically the same. In equilibrium, about 75% of both reactants were consumed. About 23% of both methyl oleate and cis-3-hexenyl acetate were converted to the self-metathesis products (9-octadecene and 3-hexene were used for GC determination, data not given in Figure 6). The rest (52%) was converted to the cross-metathesis products according to Scheme 2. It indicates the system approached statistical cross-metathesis, in accord with the characters of both reactants (classes of reactants in CM according to Grubbs [35]).

The data presented indicated that the depressed activity of HGIIN^+^Cl^−^/MCM-22 in the self-metathesis of methyl oleate was not connected with a slow diffusion of the reactant to the active centers, but most probably with the slow initiation rate. If initiation starts by coordination of the substrate molecule to the Ru atom (association and interchange mechanism [36]), the steric conditions around the Ru atom may be important. The very low initiation rate with methyl oleate may implicate some restrictions in coordination of bulky molecules; we can speculate about some confinement in the coordination sphere of Ru in HGIIN^+^Cl^−^/MCM-22 (partial immersion of HGIIN^+^Cl^−^ into support cavities and/or other deformation of the coordination sphere as a result of immobilization). When the initiation passed with *cis*-3-hexenyl acetate, the created catalytically active centers were able to ensure rapid propagation regardless the kind of substrate molecules.

## Conclusion

Hoveyda–Grubbs type metathesis catalysts with quaternary ammonium tags on NHC ligands HGIIN^+^Cl^−^ and HGIIN^+^PF_6_^−^ were immobilized on lamellar zeolites MCM-22, MCM-56, and MCM-36. Linker-free immobilizations, consisting in mixing zeolite supports with catalyst solutions and stirring the corresponding suspensions at room temperature, were successfully used. Hybrid catalysts formed (Ru content from 0.7 to 1.1 wt %) exhibited a firm attachment of Ru species to the support and high stability, which was manifested by a very low Ru leaching (from 0.1 to 0.6% of original Ru content) and possibility of catalyst reusing (five times with 99.5% conversion).

The surface stoichiometry determined from XPS indicated an ion exchange between zeolite supports (Na forms) and the Hoveyda–Grubbs type catalysts. In the case of HGIIN^+^Cl^−^, the unchanged cationic part of the Ru complex was suggested to be present in the hybrid catalyst and the counter anion, Cl^−^, was suggested to remain in the liquid phase as NaCl; however, in the case of HGIIN^+^PF_6_^−^, partial decomposition of the PF_6_^−^ anion and ligand exchange at the Ru atom most likely accompanied the immobilization. The XRD and nitrogen adsorption measurements confirmed that the layered structure of the supports was preserved in the prepared hybrid catalysts.

The activity of hybrid catalysts was studied (i) in RCM of (−)-β-citronellene and *N*,*N*-diallyl-2,2,2-trifluoroacetamide, and (ii) in self-metathesis and cross-metathesis of methyl oleate. The activity was compared with that of HGIIN^+^Cl^−^ linker-free immobilized on mesoporous molecular sieves SBA-15 (HGIIN^+^Cl^−^/SBA-15, pore diameter 6.6 nm). In RCM reactions, the activity decreased in the following order of support MCM-22 ≈ MCM-56 > SBA-15 > MCM-36. The layered structure of MCM-22 and MCM-56 most likely ensured better access of the reactants to the catalytically active centers as compared to the case of the SBA-15 based hybrid catalyst. In self-metathesis of methyl oleate, HGIIN^+^Cl^−^/SBA-15 was found to be the most active; the reaction over HGIIN^+^Cl^−^ on zeolite supports proceeded slowly and with a large induction period. In contrast to that, in the cross-metathesis of methyl oleate with *cis*-3-hexenyl acetate over HGIIN^+^Cl^−^/MCM-22, the induction period was negligible and the reaction rate slightly exceeded that over HGIIN^+^Cl^−^/SBA-15. This behavior may indicate a slow initiation by methyl oleate due to its slow coordination to the Hoveyda–Grubbs type catalysts immobilized on the zeolite supports studied.

## Experimental

### Materials and techniques

Ru alkylidene complexes HGIIN^+^Cl^−^ and HGIIN^+^PF_6_^−^ were kindly provided by Krzysztof Skowerski (Apeiron Synthesis, Wroclaw, Poland). Zeolites MCM-22, MCM-56 and MCM-36 (Na forms) were prepared according to literature [37–39] as well as mesoporous molecular sieves SBA-15 [40]. Individual supports were calcined under following conditions: MCM-22, MCM-56 in a stream of nitrogen at 482 °C for 3 h (heating rate 1 °C/min) and further after cooling down to 100 °C under air at 540 °C for 8 h with a heating rate 1 °C/min; MCM-36 under air at 540 °C for 6 h with a heating rate 2 °C/min; SBA-15 in air at 550 °C for 6 h (heating rate 1 °C/min).

Toluene (Lach-Ner) was dried for 12 h over anhydrous Na_2_SO_4_, then distilled with Na, and stored over molecular sieves type 4 Å. Dichloromethane (Lach-Ner) was dried overnight over anhydrous CaCl_2_ then distilled with CaH_2_. (−)-β-citronellene (Aldrich, purity of ≥90%), *N*,*N*-diallyl-2,2,2-trifluoroacetamide (Aldrich, 98%), *cis*-3-hexenyl acetate (Aldrich, purity ≥98%), and methyl oleate (Research Institute of Inorganic Chemistry, a.s., Czech Rep., purity of 94%, with methyl palmitate, methyl stearate, and methyl linolate being the main impurities) were used after being passed through a column filled with activated alumina.

Nitrogen adsorption/desorption isotherms were measured on a Micromeritics GEMINI II 2370 volumetric Surface Area Analyzer at liquid nitrogen temperature (−196 °C) to determine the surface area and pore volume. Prior to the sorption measurements, all samples were degassed on a Micromeritics FlowPrep060 instrument under helium at 110 °C for 6 h. X-ray powder diffraction (XRD) data were obtained on a Bruker AXS D8 Advance diffractometer with a graphite monochromator and a Vantec-1 position sensitive detector using Cu Kα radiation (at 40 kV and 30 mA) in Bragg−Brentano geometry.

The X-ray photoelectron spectra (XPS) of the samples were measured using a modified ESCA 3 MkII multitechnique spectrometer equipped with a hemispherical electron analyzer operated in a fixed transmission mode. Al Kα radiation (1486.6 eV) was used for electron excitation. The binding energy scale of the spectrometers was calibrated using the Au 4f_7/2_ (84.0 eV) and Cu 2p_3/2_ (932.6 eV) photoemission lines. The pressure of residual gases in the analysis chamber during spectra acquisition was 6 × 10^−9^ mbar. The powder samples were spread on an aluminum surface. The spectra were measured at room temperature and collected at a detection angle of 45° with respect to the macroscopic sample surface plane. Survey scan spectra and high-resolution spectra of overlapping Ru 3d + C 1s photoelectrons, and N 1s, Cl 2p, P 2s, and F 1s photoelectrons were measured. The spectra were curve-fitted after subtraction of the Shirley background [41] using the Gaussian−Lorentzian line shape and the damped nonlinear least-squares algorithms (software XPSPEAK 4.1) [42]. The quantification of elemental concentrations was accomplished by correcting integrated intensities of photoelectron peaks for the transmission function of the electron analyzer and the pertinent photoionization cross sections [43]. In the calculations, a homogeneous composition of the analyzed layer of the measured samples was assumed. The typical error for the quantitative analysis by XPS was approximately 10% [44].

The determination of the ruthenium content was performed by inductively coupled plasma mass spectrometry (ICP–MS) by the Institute of Analytical Chemistry (ICT, Prague, Czech Republic).

### Catalyst preparation

Immobilization of HGIIN^+^X^−^ complexes was performed by stirring a mixture of complex and support in CH_2_Cl_2_ at room temperature (3 h) under argon atmosphere. Details are given elsewhere [21]. For immobilization, calcined (dehydrated) supports (300 °C, 3 h) were used. The amount of support, Ru complex submitted and Ru content in hybrid catalyst are given in Table 4. Catalyst prepared by immobilization of HGIIN^+^Cl^−^ on MCM-22 was labelled as HGIIN^+^Cl^−^/MCM-22; other catalysts were labelled in a similar way.

**Table 4:** Amounts of support and HGIIN^+^X used for preparation of hybrid catalysts.

Catalyst	Weight of support (mg)	Weight of HGIIN^+^X^−^ (mg)	Ru content in catalyst (wt %)	*f*^ a^

HGIIN^+^Cl^−^/MCM-22	250	23.9	1.1	0.97
HGIIN^+^PF_6_^−^/MCM-22	140	17.8	0.9	0.66
HGIIN^+^Cl^−^/MCM-56	305	30.2	1.1	0.99
HGIIN^+^Cl^−^/MCM-36	835	87.0	0.7	0.54
HGIIN^+^Cl^−^/SBA-15	339	36.5	1.2	0.99

^a^*f* = fraction of Ru attached to the support.

### Testing of catalyst activity

Metathesis reactions were performed under Ar atmosphere in Schlenk tubes equipped with magnetic stirring bars. In a typical RCM experiment the amount of catalyst corresponding to 1 μmol of Ru was put into the reactor, then toluene (13 mL) was added and the suspension was heated to 60 °C. The reaction was started by addition of (−)-β-citronellene (2 mmol) under stirring (900 rpm). At given time intervals, samples (0.1 mL) were taken and quenched with ethyl vinyl ether, and after centrifugation, the supernatants were analyzed by gas chromatography (GC). In the cross-metathesis experiment, a mixture of methyl oleate (0.25 mmol) and *cis*-3-hexenyl acetate (0.25 mmol) was added to the suspension of catalyst (1 μmol of Ru) in toluene (1.7 mL) at 30 °C under stirring. The sampling, quenching and analysis steps were performed similarly as for RCM of (−)-β-citronellene.

A high-resolution gas chromatograph (Agilent model 6890) with a DB-5 column (length of 50 m, inner diameter of 320 μm, stationary phase thickness of 1 μm) equipped with FID detector was used for reaction product analysis. Temperature programs were: (i) from 80 °C to 260 °C with ramp 20 °C/min for (−)-β-citronellene products, and (ii) from 80 °C to 325 °C with ramps 5 °C and 20°C for methyl oleate and DAF products. Retention times (in min) were 8.63 (citronellene), 4.88 (methylcyclopentene), 19.3 (DAF), 19.7 (*N*-(2-trifluoroacetyl)-2,5-dihydropyrrole), 41.3 (methyl oleate), 33.9 (octadecene), and 57.5 (diester). *n*-Nonane was used as an internal standard, whenever required. Individual products (all are known compounds) were identified by gas chromatography and mass spectrometry (GC−MS) (ThermoFinnigan, FOCUS DSQ II Single Quadrupole). The absolute error in the determination of conversion was ±2%.

## Supporting Information

File 1 XRD patterns of catalysts and supports, conversion curves for self-metatheses of methyl oleate and *cis*-3-hexenyl acetate, splitting experiment.
