# Supplementary material for: Ru complexes of Hoveyda–Grubbs type immobilized on lamellar zeolites: activity in olefin metathesis reactions
Source: Beilstein J Org Chem. 2015 Nov 4;11:2087–96. doi: 10.3762/bjoc.11.225 (PMC4660987; doi:10.3762/bjoc.11.225)
Supplement: File 1 — XRD patterns of catalysts and supports, conversion curves for self-metatheses of methyl oleate and cis-3-hexenyl acetate, splitting experiment. [file Beilstein_J_Org_Chem-11-2087-s001.pdf]

Supporting Information

for

Ru complexes of Hoveyda–Grubbs type immobilized on lamellar zeolites: activity in olefin metathesis reactions

Hynek Balcar*, Naděžda Žilková, Martin Kubů, Michal Mazur, Zdeněk Bastl and Jiří Čejka

Address: J. Heyrovský Institute of Physical Chemistry, Academy of Sciences of the Czech Republic, v.v. i. Dolejškova 2155/3, 182 23 Prague 8, Czech Republic

Email: Hynek Balcar* - hynek.balcar@jh-inst.cas.cz

* Corresponding author

XRD patterns of catalysts and supports, conversion curves for self-metatheses of methyl oleate and *cis*-3-hexenyl acetate, splitting experiment

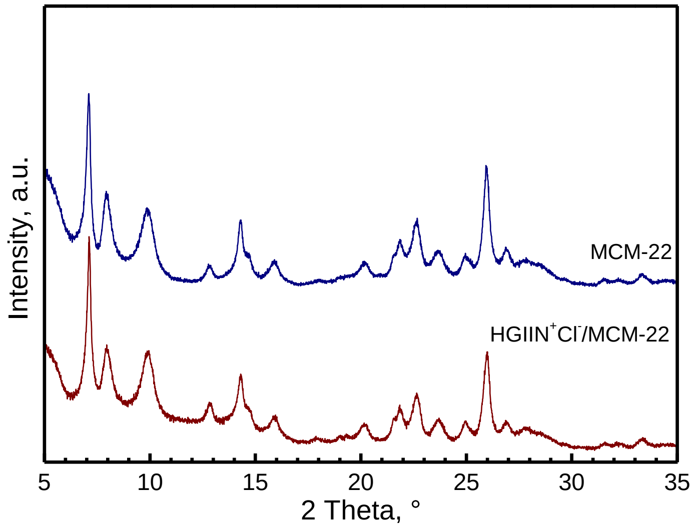

Figure S1: XRD patterns of parent MCM-22 and HGIIN^{+}Cl^{-}/MCM-22 catalyst.

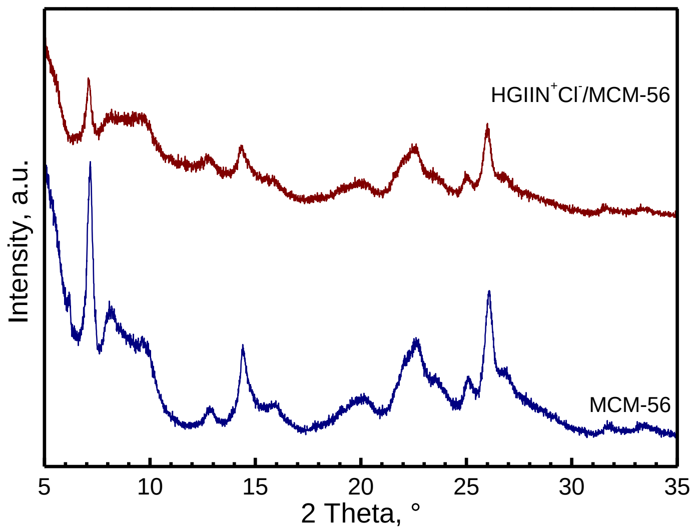

Figure S2: XRD patterns of parent MCM-56 and HGIIN^{+}Cl^{-}/MCM-56 catalyst.

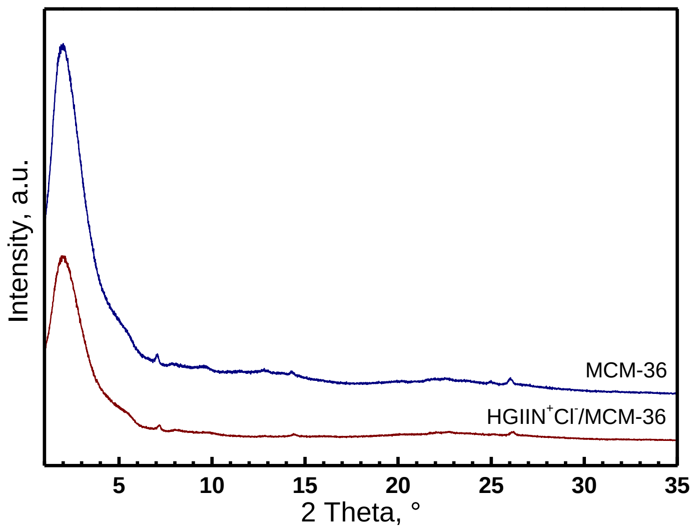

Figure S3: XRD patterns of parent MCM-36 and HGIIN^{+}Cl^{-}/MCM-36 catalyst.

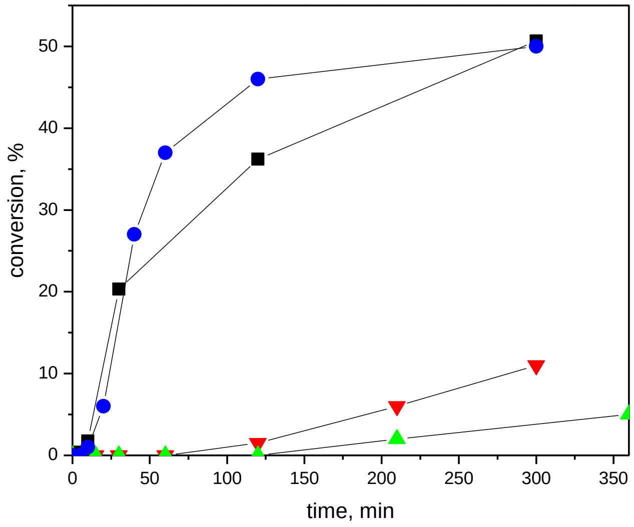

Figure S4: Self-metathesis of methyl oleate over HGIIN$^+$Cl$^-$ (●), HGIIN$^+$Cl$^-$/SBA-15 (■), HGIIN$^+$Cl$^-$/MCM-22 (▼) and HGIIN$^+$Cl$^-$/MCM-56 (▲). Toluene (toluene+CH_2Cl_2, v/v = 78/22 for (●)), 30 °C, molar ratio oleate/Ru=250, c_{ol} = 0.15 mol/L.

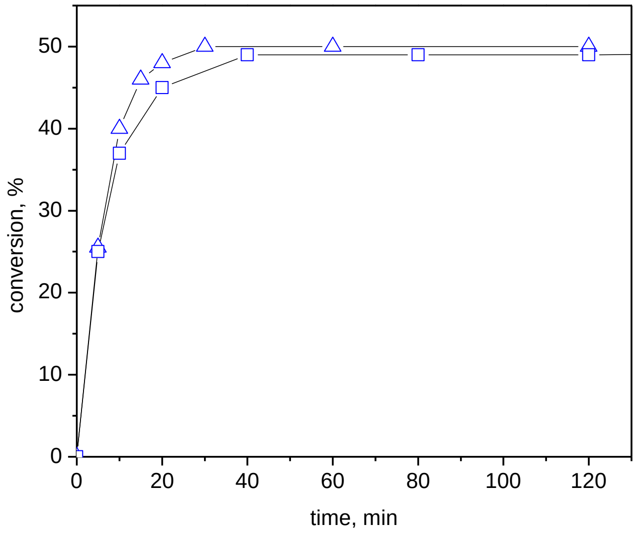

Figure S5: Self-metathesis of *cis*-3-hexenyl acetate over HGIIN$^+$Cl$^-$/MCM-22 (Δ) and HGIIN$^+$Cl$^-$/SBA-15 (□). Toluene, 30 °C, molar ratio 3-hexenyl acetate/Ru= 250, c_{ac}=0.15 mol/L.

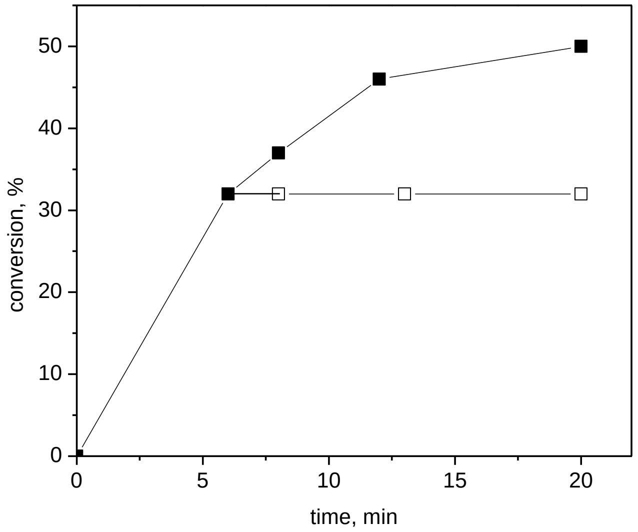

Figure S6: Splitting test for self-metathesis of cis-3-hexenyl acetate over $HGIIN^{+}Cl^{-}$/MCM-22. Toluene, 30 °C, molar ratio *cis*-3-hexenyl acetate/Ru= 2000, c_{ac}= 0.15 mol/L. Heterogeneous system (■), system without heterogeneous catalyst (□).
